# CaRDS - the statewide California Residential water Demand and Supply open dataset

**DOI:** 10.1038/s41597-024-03474-y

**Published:** 2024-06-14

**Authors:** Marie-Philine Gross, Alvar Escriva-Bou, Erik Porse, Andrea Cominola

**Affiliations:** 1 Chair of Smart Water Networks, Technische Universität Berlin, Berlin, 10623 Germany; 2 Einstein Center Digital Future, Berlin, 10117 Germany; 3 Civil and Environmental Engineering and Institute of the Environment and Sustainability, University of California, Los Angeles, CA 90095-1496 USA; 4 California Institute for Water Resources, University of California Agriculture and Natural Resources, Davis, CA 95618 USA

**Keywords:** Hydrology, Water resources

## Abstract

As water scarcity becomes the new norm in the Western United States, states such as California have increased their efforts to improve water resilience. Achieving water security under climate change, population growth, and urbanization requires an integrated multi-sectoral approach, where adaptation strategies combine supply and demand management interventions. Yet, most studies consider supply-side and demand-side management strategies separately. Water conservation efforts are mainly driven by policy requirements and publicly available data to assess the effectiveness of demand- and supply-side management policies is often hard to find and unstructured. Here we present CaRDS - the statewide California Residential water Demand and Supply open dataset. CaRDS encompasses nine years (2013-2021) of monthly water supply and demand time series for 404 water suppliers in California, USA, compiled from different open-access data sources. Access to detailed temporal and spatial water supply operations and demands at the state-level can be useful to researchers and practitioners to realize applications such as evaluating the effectiveness of water conservation policies and discovering regional differences in water resilience measures.

## Background & Summary

In recent decades, droughts have become increasingly frequent and prolonged in the Western United States^1^. Climate change is expected to exacerbate the severity of these extreme hydroclimatic events, putting more stress on the already scarce water resources in the region^2,3^ and increasing the vulnerability of water systems^4^. National and regional governments worldwide are implementing new multi-sectoral adaptation strategies and policies to adapt to climate variability and change in the pursuit of water security^5^. In response to mid-twentieth century water scarcity, the federal, state, and local governments in California built large-scale infrastructure to move water over long distances for use by cities and farms^6–8^. As new sources dwindled, the drought in 1976-77 instigated drought restrictions, which began several decades of state and federal policies to reduce water use through building codes and efficiency standards, especially for indoor use^9^. To counter the more severe droughts in recent times and in the future, the California State Legislature enacted two policy bills in 2018 to establish a new foundation for long-term water conservation and drought adaption planning - “Making Water Conservation a California Way of Life”^10,11^. Water retailers will need to implement water demand management and conservation strategies to meet goals depending on local characteristics of efficiency investments, landscape irrigation, and land use characteristics^12^.

Water suppliers have addressed water scarcity challenges through a mix of supply and demand-side measures. Water supply strategies focus on increasing water availability by developing new sources (e.g., surface supplies, groundwater, water reuse, and desalination) or by optimizing the management of existing sources. Water demand-side management focuses more on the consumers and their water use. Active areas of research are among others water demand forecasting, water consumption behavior change programs (e.g., via feedback on end-use water use activities or fixture upgrades), alternative water pricing schemes, and leak management. Existing literature generally considers demand-side management and supply-side measures separately, whereas the concept of integrated water management recommends shifting from an isolated view on certain parts of water management and the water cycle to a more holistic approach.

A chance to realize such a multi-sectoral management strategy lies within the availability of FAIR (findable, accessible, interoperable, and reusable) data^13^. The deployment of digital technology and sensors in the water sector sparked the interest of researchers and practitioners towards an unprecedented amount of highly disaggregated data in time and space and related data driven analytics for both water supply and demand modelling and optimization^14^. A recent study showed that the digitalization of water utilities is globally underway, but its progress is highly dependent on individual management decisions^15^. This means big water data is often only available for more progressive water utilities and cannot be shared with the public due to security concerns. On the other hand, government organizations try to increasingly make public data more accessible by releasing data online for everyone to use. Most of the time, however, this data is not directly usable for analysis purposes^16^. Available data often lacks documentation, has inconsistent temporal resolution, or has machine-readability issues. This then requires extensive data processing, hampering a direct use of accessible data sources. For the reasons above, a large-scale, California-wide evaluation of the trade-offs of water supply and demand management strategies to support large-scale planning efforts for demand management in combination with supply-side interventions is not readily available.

Here we present CaRDS - the statewide *California Residential water Demand and Supply* open dataset, which contains monthly values of water supply and residential water demand disaggregated at the supplier level for 404 water suppliers in California from 2013 to 2021. CaRDS advances the literature in three ways: (i) data usability - this dataset closes the gap between data availability and the direct usability of public water supply and demand data sets; (ii) data consistency - CaRDS integrates continuous time series of monthly records for water supply and demand, together with corresponding data on precipitation and drought conditions for each supplier location. CaRDS thus gathers data that are currently scattered in various sources and reported with different formats, time/space aggregation levels, and units, making them consistent; (iii) data coverage - the dataset provides a consistent multi-annual statewide coverage of water suppliers in California, allowing research and applications that go beyond individual suppliers/regions and short-term analysis. CaRDS can be used to study the trade-offs between water supply and residential demand at the state scale, as well as to support regional studies, e.g., at the county, climatic zone, or hydrologic region scale. Further, water suppliers can compare their management strategies with neighboring suppliers. CaRDS can easily be extended with new data each year and it is possible to combine it with other data sets, e.g., for electricity or wastewater.

**Fig. 1:**
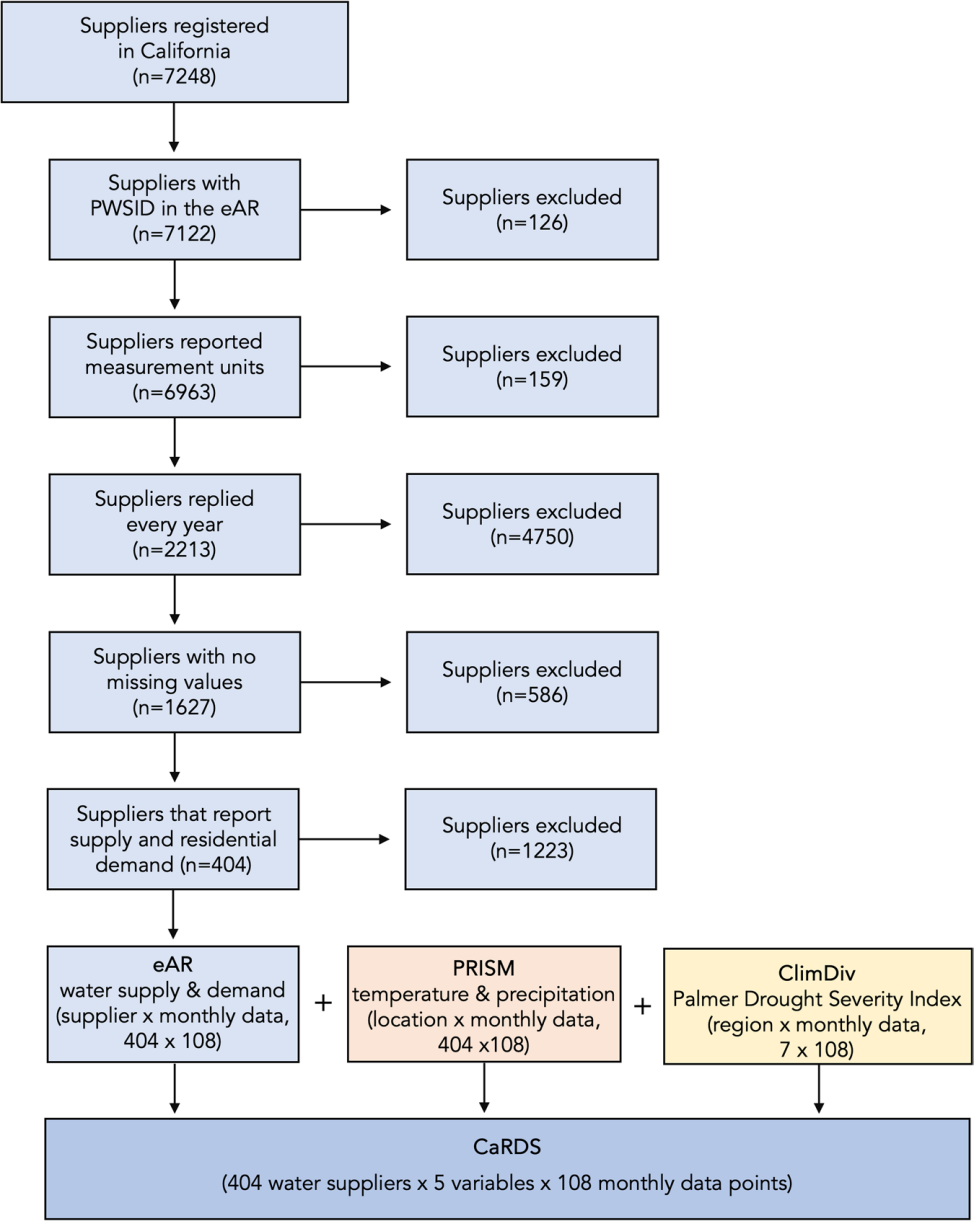
Flow diagram with exclusion criteria and relative data records excluded/retained in the creation of CaRDS. The flow diagram reports the exclusion criteria applied to the data retrieved from the Electronic Annuals Report (eAR) from the State Water Resources Control Board. The flow diagram is adapted from the Preferred Reporting Items for Systematic Reviews and Meta-Analyses guidelines (PRISMA Flow Diagram^25^).

CaRDS is compiled based on three different public data sources: the Electronic Annual Reports (eAR) from the California State Water Resources Control Board^17^, the PRISM Climate Data from the PRISM Climate Group^18^, and the Climate Division Data (ClimDiv) from National Oceanic and Atmospheric Administration^19^. First, we extract water supplier information, the corresponding monthly water supply and residential demand from the eARs, and perform pre-processing steps to improve data consistency and enable data integration. We then use this information to geographically match the water suppliers with different climatic variables from PRISM and ClimDiv (see Fig. 1). In the following sections we describe CaRDS, the data processing steps, and its technical validation in more detail. Finally, we demonstrate how we can make open-access data workable.

## Methods

CaRDS merges data from three different open-access sources to provide a temporally and spatially detailed description of water supply and residential water demand in California at the supplier level, along with hydroclimatic information for each location. In the following sections, we will first introduce the original data sources separately and explain the processing steps we performed for each type of data. We will then describe the data integration of the different data sources into one comprehensive dataset, CaRDS.

### Electronic Annual Report

At the core of CaRDS are the water demand and supply data, which are extracted from the eAR. The eAR is a mandatory annual survey conducted by the State Water Resources Control Board (SWB) of California. As early as the 1980’s, urban water agencies in California submitted detailed monthly water use data to the California Department of Water Resources. According to the California Water Law, the correct term to refer to such urban water agencies partaking in the data reporting would be *community water systems*. Here we will utilize the more commonly used term *water suppliers*. Over the course of decades, the frequency and detail of reporting varied. The SWB moved to an online survey in 2009, giving it today’s name eAR. Starting in 2013, suppliers were required to submit detailed monthly data as part of drought management requirements, and the regular collection of detailed operations data was standardized as part of duties of the SWB. In 2019 the SWB released online the completed eARs starting from 2013. Here we use the online available surveys from 2013 to 2021^17^, in total nine years, to obtain information about water supply and demand at the supplier level for the whole state of California.

The reports encompass 3306 coded variables in the most current version, impeding a direct extraction of variables of interest. Additionally, the data quality is impacted by human error as data are manually reported by each water suppliers. This results in missing values, fluctuating reporting rates (despite the mandatory nature of the reporting), and an inconsistent encoding of string characters, which makes machine readability challenging. These data quality issues have greatly limited the past use of this data outside of the SWB.

For each year, we extract the following seven variables: supplier ID, monthly water production (supply), measurement unit of the water supply, total and residential monthly water deliveries (demand), measurement unit of water demand, and number of people served. Suppliers often also report agricultural, commercial, and institutional water demands, which are not included in CaRDS, as it focuses on residential water demand only. One of the challenges in data cleaning is that variable name conventions were changed by the SWB starting with the 2020 survey. Therefore, we process the survey data in two groups separately, one including the entries reported between 2013 and 2019, the second comprising those reported between 2020 and 2021. Another challenge is that the number of suppliers varies heavily between the different reporting years, ranging from 4000 to 7000 answers. To account for continuous reporting and create a reliable dataset with consistent multi-annual time series of data for each supplier, we apply the exclusion criteria shown in Fig. 1, where criteria 1 to 4 are evaluated separately on the water supply and demand time series. A supplier is excluded while compiling CaRDS if:

1. they do not report a unique identifier, i.e., the Public Water System Identification Number (PWSID), in their annual reporting.
2. they never report the unit for water supply and demand in the study period.
3. they do not report every year during the study period.
4. their report has one or more missing water supply or demand (total and residential) values during the study period.
5. they do not report water supply and water demand for residential use.

Missing or inconsistent information in other selected variables (i.e., measurement units for supply, measurement units for demand, and people served) is not a reason to exclude suppliers. Missing population data is interpolated by using the previously reported value, as population numbers reported by the water suppliers are very stable. The last step to create a consistent dataset is converting water supply and demand values into standard measurement units according to the International System of Units. Water suppliers often report different units for water supply and demand, thus we convert them separately. Additionally, starting in 2020 there is no information on measurement units for all water suppliers. We solve this problem by applying the following two unit conversion steps:

1. Compute the difference in magnitude to January 2019
  1 $$\begin{array}{r}{\Delta }_{i,N}=\frac{{X}_{i,Jan19}}{{X}_{i,JanN}},\end{array}$$
  with *X* being the reported value for *i* = {Supply, Demand} and *N* = {2020, 2021}.
2. Assign measurement units and convert to standard units.
  2 $$\widehat{{X}_{i,n}}=\left\{\begin{array}{ll}{X}_{i,n}, & \,\mathrm{if}\,\,{\Delta }_{i,N} < 600\,(\mathrm{Values\ reported\ in\ Gallons}),\\ {X}_{i,n}\ast 748, & \,\mathrm{if}\,\,{\Delta }_{i,N}\,600 < 3,000\,(\mathrm{Values\ reported\ in\ CCF}),\\ {X}_{i,n}\ast 325,851, & \,\mathrm{if}\,\,{\Delta }_{i,N}\,3,000 < 500,000\,(\mathrm{Values\ reported\ in\ AF}),\\ {X}_{i,n}\ast 1{0}^{6}, & \,\mathrm{if}\,\,{\Delta }_{i,N} > 500,000\,(\mathrm{Values\ reported\ in\ Mio.\ Gallons}).\end{array}\right.$$
  with *n* being the monthly value we need to convert.

To ensure the unit conversion is successful, we analyze the temporal consistency of the time series by means of outlier detection (see Section “Consistency of time series and potential outliers").

### PRISM Climate Data

To account for hydroclimatic influences and conditions on water management strategies, we include the monthly mean temperature and cumulative precipitation for the service area of each supplier in our dataset. The PRISM Climate Group^18^ gathers climatic data from various sources, applies quality control mechanisms, and releases various climate datasets with multiple spatial and temporal resolutions for the USA. The coordinates for each supplier’s location are needed to obtain the related climatic data. Based on the ZIP code of a supplier we compute the spatial centroid for its service area and use the centroid coordinates as input for the PRISM data retrieval. This way we could match all supplier locations with the exception of two that were added manually. With the batch retrieval we compute a mean monthly temperature (in Celsius) and the cumulative monthly precipitation (in Millimeters) for each supplier location.

### ClimDiv

Given the historical importance and environmental and socio-economic impacts of multi-year droughts in California, we include the Palmer Drought Severity Index (PDSI)^20^ as an additional hydroclimatic factor in CaRDS. PDSI is a measure to estimate relative dryness and it is very effective in accounting for long-term drought conditions, taking the basic effects of global warming into account. The PDSI is provided by NOAA and calculated for large areas^19^, roughly following the division of hydrologic regions. NOAA divides California into seven climate divisions, instead of the ten regions considered by the SWB. This is achieved by either merging two hydrologic regions together (San Joaquin River and Tulare Lake; South Lahontan and Colorado River) or splitting one region before merging (Central Coast is split between San Francisco Bay and South Coast). We retrieve monthly PDSI values for each of the seven divisions for the study period of nine years and match them to each water supplier based on ZIP Codes.

### Data integration and compilation of CaRDS

After data processing on the three individual datasets presented in the previous sections, we integrate them and compile CaRDS (see Fig. 1). Each time series we extract is linked to the unique PWSID, making it easy to merge the data and have a consistent set of water supply/demand and hydroclimatic variables for each supplier. The version of CaRDS released with this publication includes 404 water suppliers, each with five corresponding time series of the following monthly variables: water supply, water demand, mean temperature, cumulative precipitation, and PDSI. Each variable has a length of 108 time steps, covering in total nine years in monthly intervals. A detailed overview of the variables included in CaRDS, along with a short description and their units, is provided in Table 1.

**Table 1:** Overview of the variables included in CaRDS.

Variable name	Description	Unit
PWSID	Public Water System Identification Number	—
Supply	Monthly water produced by supplier	*gal*
Demand	Monthly water sold by supplier to residential customers	*gal*
Mean Temperature	Average monthly temperature in supplier location	^°^*C*
Precipitation	Cumulative monthly precipitation in supplier location	*mm*
PDSI	Palmer Drought Severity Index in supplier climatic division	—

## Data Records

The CaRDS^21^ dataset is available on HydroShare and can be accessed via the following link: 10.4211/hs.4ec7019fe63944bf87d40d2cdfa0d686. The data is structured by two levels of key identifiers. The first level is the unique supplier identification number PWSID and the second level contains the time-series of monthly water supply, demand, mean temperature, cumulative precipitation, and PDSI (see Table 1). In the same repository we also share a file called *Supplier_Info.csv*, which provides secondary information about the suppliers in our dataset. This file mostly contains geographic information (ZIP code, county, hydrologic zone, climatic zone, and climatic division), along with information on the population served and the size of each supplier.

## Technical Validation

**Fig. 2:**
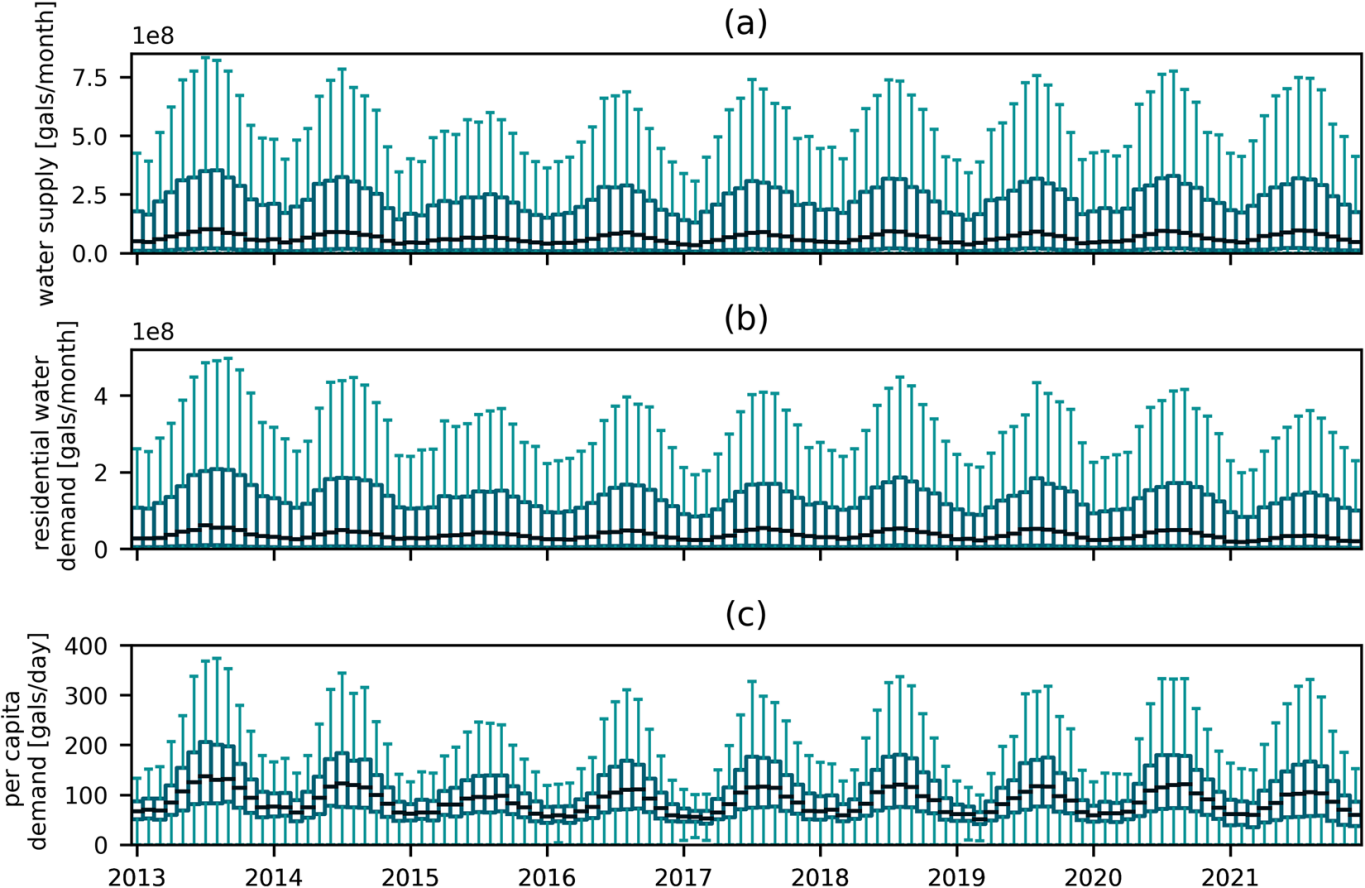
Time series of (**a**) water supply, (**b**) residential water demand, and (**c**) daily per capita water consumption for all water suppliers included in CaRDS. The line in the middle of each box represents the median value across all suppliers, boxes mark the first and third quartile. The length of box whiskers is equal to 1.5 times the interquartile range. In all three instances a distinct seasonal pattern emerges, with higher values during the summers. This behavior is expected as California overall has wet winters and dry summers. Further, there is a noticeable smaller peak in all three instances in the summer of 2015. Mandatory water conservation measures were in place in California during this period to overcome the ongoing drought. Data in CaRDS thus capture climate-driven water management and policy dynamics.

As the CaRDS dataset we present here is largely based on survey data, using traditional approaches for data validation by modeling the retrieved data or comparing it to similar datasets is not possible. A way to check the validity and plausibility of the water supply and residential demand time series is to look at their patterns. In Fig. 2, we display the monthly distribution of the water supply and demand for all suppliers over the nine years included in CaRDS, as well as the computed daily per capita water use. For all three instances a distinct seasonal pattern emerges, with higher values during the summer periods. This behavior is expected as California overall has wet winters and dry summers. Further, there is a noticeable smaller peak in all three instances in the summer of 2015. Water scarcity and policy decisions resulted in establishment of mandatory water conservation measures in California to overcome the ongoing drought during that period. This implies that our dataset is able to reasonably and plausibly capture both the seasonal nature of its variables, and the influence of water management dynamics.

### Consistency of time series and potential outliers

**Fig. 3:**
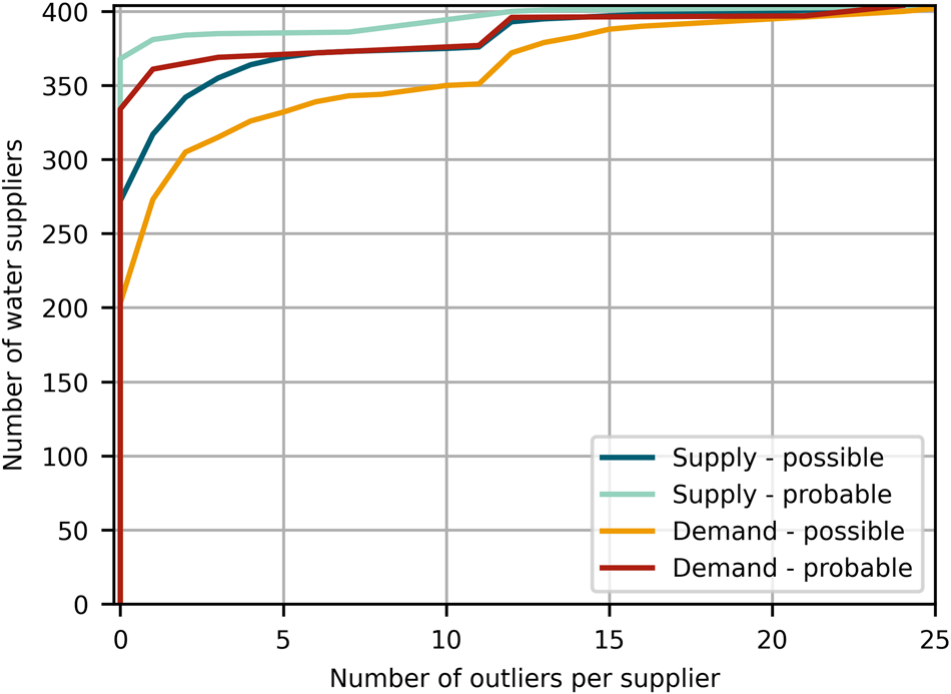
Cumulative distribution of possible and probable outliers per water supplier by applying Tukey’s fences to each water supply and demand time series in CaRDS.

To consolidate and assess our data processing methodology, in particular the conversion of measurement units, we apply Tukey’s fences^22^ separately for the water supply and demand time series of each supplier. By using interquartile ranges, we can identify possible and probable outliers for each time series. We find that 32% of the supply time series and 49% of the demand time series have possible outliers, while probable outliers exist in 9% and 17% of the supply and demand time series, respectively. These values are non-negligible. However, the distributions in Fig. 3 show that most water suppliers exhibit no outliers and an additional 4% (supply) and 20% (demand) exhibit only between 1 and 5 outliers in their monthly supply or demand values. We detect a small incline around 12 detected outliers in the cumulative distributions in Fig. 3 across all classified outliers for supply and demand. This can indicate that the measurement units might have been wrongly reported for one full year by only 10% of the suppliers. Possible and probable outliers in water supply and demand data are thus expected to only marginally influence the quality of data for individual suppliers (i.e., there are no suppliers with major portions of outliers in their data time series). Overall, the dataset encompasses two climatic extreme events, where outlier values are expected to a certain degree. Further, the computation of missing measuring units is not exact, but rather an approximation based on empirical value ranges. Nevertheless, a deviation in value magnitude between 2019 and 2020 is only detected for 4% of the suppliers underlining the validity of the approach.

Aiming for an automated and general way of pre-processing the different data sources without resource-intensive and subjective manual cleaning, and considering the qualitative challenges of the data collection, we decided not to exclude the potential outliers from the CaRDS dataset we are releasing with this publication. We thus did not exclude any further outliers from CaRDS after application of the initial exclusion criteria (Fig. 1), to preserve the original data structure and to give future users the possibility to rely on as many data as possible and optionally remove further data depending on their specific research needs. To further study the nature of each outlier and possibly remove some of them in case some applications based on CaRDS require it, an in-depth analysis of each time series may be necessary. More advanced outlier detection for time series data can rely on Autoregressive Integrated Moving Average (ARIMA)^23^ models or unsupervised clustering such as DBSCAN^24^, but further outlier detection is out of the scope of this study.

### Analysis of population served

**Fig. 4:**
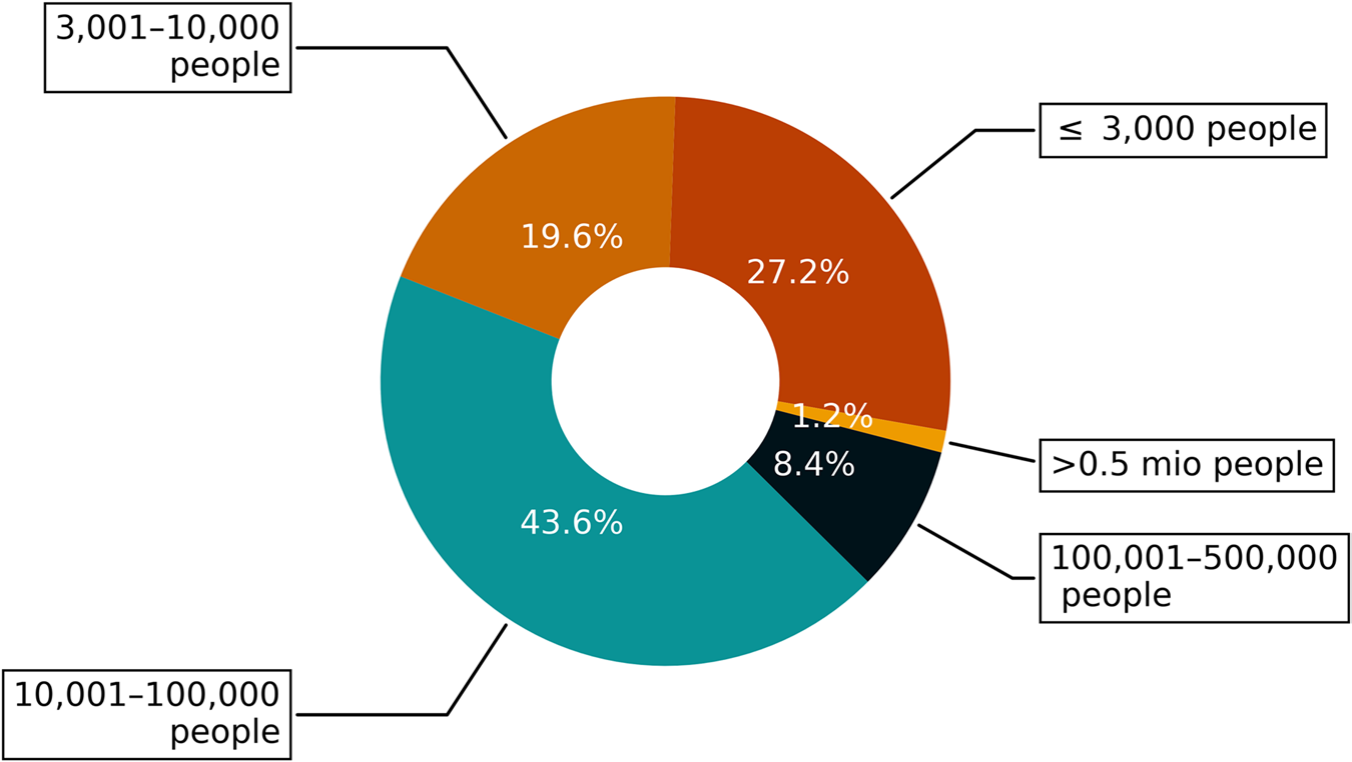
Relative proportion of water suppliers included in CaRDS across different utility sizes. Utility size is quantified as the population served by each supplier.

We analyze the population that is served by the water suppliers included in CaRDS to demonstrate that the CaRDS dataset is overall representative of the state of California. The water suppliers in CaRDS serve 52% (20. mio.) of the population in California. We further investigate the size of the communities that are served by the water suppliers (see Fig. 4). We see that nearly 50% of suppliers serve communities smaller than 10,000 people, representing small towns or neighborhoods and very rural settlements. The other half of the suppliers serve medium-sized towns and big cities, representing the more urbanized and metropolitan areas of California. CaRDS therefore represents well small and medium-sized suppliers, while large suppliers, e.g., Urban Water Retail Suppliers (URWS), are underrepresented. If the aim is to investigate URWS only, other data sources such as the SWB water conservation portal may be more suitable. A detailed overview of how many people are served by hydrologic region and climatic zone in California can be found in Table 2.

**Table 2:** Distribution of water suppliers and corresponding population served in the 16 climate zones and 10 hydrologic regions in California.

Climate Zone			Hydrologic Region		
Zone ID	Supplier count	Served population	Hydrologic region	Supplier count	Served population
1	15	174,159	North Coast	33	441,043
2	26	405,755	Sacramento River	35	954,708
3	29	5,654,892	North Lahontan	8	50,862
4	11	525,014	San Francisco Bay	26	6,221,284
5	22	333,704	San Joaquin River	36	912,626
6	20	901,753	Central Coast	50	1,026,902
7	5	451,599	Tulare Lake	24	1,041,544
8	33	2,774,847	South Lahontan	36	653,322
9	53	1,989,615	South Coast	140	8,603,622
10	31	2,668,344	Colorado River	16	214,241
11	16	348,472			
12	51	1,841,846			
13	21	1,084,999			
14	37	671,396			
15	9	172,470			
16	25	121,289			

The climatic zones are based on the zones defined by the Building Energy Efficiency Standards by the California Energy Commission.

### Spatial analysis

**Fig. 5:**
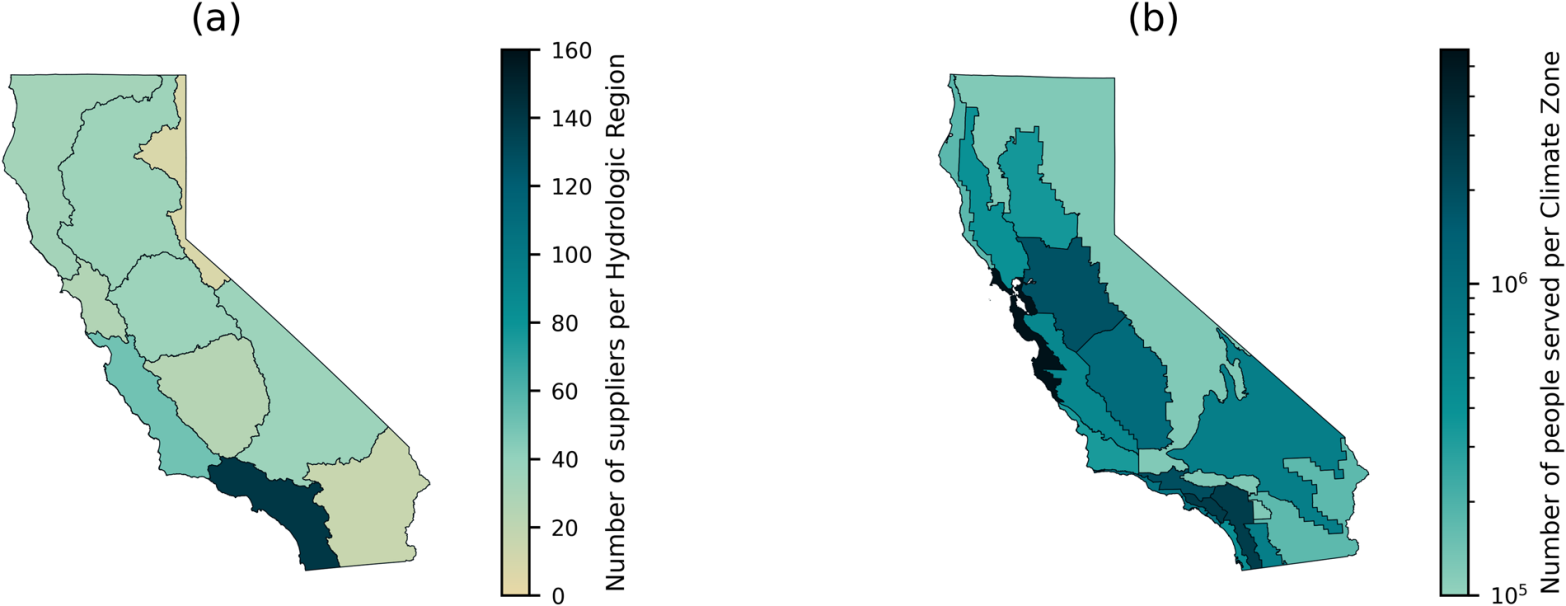
Spatial distribution of water suppliers included in CaRDS. The count of water suppliers located in each of the 10 Hydrologic Regions in California is reported in (**a**). The amount of people served by these suppliers in each of the 16 Climate Zones is represented in (**b**).

To further verify the spatial representation of CaRDS, we present different spatial distributions of the suppliers in the state of California. To demonstrate that the dataset achieves a satisfactory representation of water suppliers in California, Fig. 5(a) shows the number of suppliers of CaRDS in each of the 10 hydrologic regions California is divided in. We see that, first, each hydrologic region is represented in the dataset. Second, urbanized areas of the state are reflected with a higher number of suppliers being in metropolitan regions (South Coast and San Francisco Bay), and fewer suppliers in rural areas or areas further from metropolitan centers and core infrastructure (North Lahontan and Colorado River). Figure 5(b) shows a similar spatial distribution for the amount of customers served by the suppliers in CaRDS based on the 16 climatic zones in California. A detailed overview of the number of suppliers and population served per hydrologic region and climate zone can be found in Table 2.

## Usage Notes

Data users should take into account the assumptions we made in creating the dataset. For water supply, eAR records do not directly report the monthly water that is supplied to customers, but the water that is produced in a month. Water production means water is either treated surface or ground water, bought from another supplier, or reused and then introduced into the water suppliers’ network through direct distribution or storage. The storage component means that the data might include time lags for when water is actually supplied. Further, the eAR does not report the actual demands of households, but the amount of water that suppliers billed to residential customers. This means that the recorded water demand is dependent on the water meter/reading resolution and meter reading accuracy. In California, most larger urban water suppliers have metered data, but some smaller suppliers may use non-metering methods to quantify customer water use. As socio-economic factors become more important for water management research, the provided supplementary information of the suppliers (see Data Records) gives information on the population served by each supplier. Further, the provided ZIP codes can be used to cross-correlate the data in CaRDS with those from the U.S. Census Bureau. There is a wealth of socio-economic data already available at different spatio-temporal resolutions and it is well organized in their public available data repository (census.gov).

## Data Availability

The data pre-processing leading to the developement of CaRDS is based on open source Python software. Jupyter Notebooks with the code to pre-process, transform, and merge the different data sources reported in this article are available on HydroShare^21^ at 10.4211/hs.4ec7019fe63944bf87d40d2cdfa0d686.

## References

[1] Zhang F (2021). Five decades of observed daily precipitation reveal longer and more variable drought events across much of the western United States. Geophysical Research Letters.

[2] Greve P (2018). Global assessment of water challenges under uncertainty in water scarcity projections. Nature Sustainability.

[3] Vicuna S, Maurer EP, Joyce B, Dracup JA, Purkey D (2007). The sensitivity of California water resources to climate change scenarios 1. JAWRA Journal of the American Water Resources Association.

[4] Fu X, Tang Z (2013). Planning for drought-resilient communities: An evaluation of local comprehensive plans in the fastest growing counties in the US. Cities.

[5] Furlong C, Brotchie R, Considine R, Finlayson G, Guthrie L (2017). Key concepts for integrated urban water management infrastructure planning: lessons from Melbourne. Utilities Policy.

[6] Mitchell, D. *et al*. Building drought resilience in California’s cities and suburbs. *Public Policy Institute of California* 1–49 (2017).

[7] Cahill R, Lund J (2013). Residential water conservation in Australia and California. Journal of Water Resources Planning and Management.

[8] Hanak, E.* Managing California’s water: From conflict to reconciliation* (Public Policy Instit. of CA, 2011).

[9] Quinn, T. Forty years of California water policy: What worked, what didn’t and lessons for the future (2019).

[10] California State Assembly. Assembly bill no. 1668. https://leginfo.legislature.ca.gov/faces/billTextClient.xhtml?bill_id=201720180AB1668 (2018).

[11] California State Senate. Senate bill no. 606. https://leginfo.legislature.ca.gov/faces/billTextClient.xhtml?bill_id=201720180SB606 (2018).

[12] California State Water Resources Control Board. Water conservation portal. https://www.waterboards.ca.gov/water_issues/programs/conservation_portal/conservation_reporting.html Accessed on 21.11.2023 (2023).

[13] Wilkinson MD (2016). The fair guiding principles for scientific data management and stewardship. Scientific data.

[14] Zounemat-Kermani M (2020). Neurocomputing in surface water hydrology and hydraulics: A review of two decades retrospective, current status and future prospects. Journal of Hydrology.

[15] Daniel I (2023). A survey of water utilities’ digital transformation: drivers, impacts, and enabling technologies. npj Clean Water.

[16] Stagge JH (2019). Assessing data availability and research reproducibility in hydrology and water resources. Scientific data.

[17] California State Water Resources Control Board. Electronic annual report. https://www.waterboards.ca.gov/drinking_water/certlic/drinkingwater/ear.html Accessed on 21.04.2023 (2023).

[18] PRISM Climate Group, Oregon State University. PRISM time series data. https://prism.oregonstate.edu Accessed on 05.07.2023 (2023).

[19] National Oceanic and Atmospheric Administration. Historical palmer drought severity indices. https://www.ncei.noaa.gov/pub/data/cirs/climdiv/ Accessed on 27.06.2023 (2023).

[20] Palmer, W. C.*Meteorological drought*, vol. Res. Paper No.45 (US Department of Commerce, Weather Bureau, 1965).

[21] Gross M, Escriva-Bou A, Porse E, Cominola A (2024). HydroShare.

[22] Tukey, J. W.*Exploratory data analysis* (Addison-Wesley Publishing Company, 1977).

[23] Box, G. E., Jenkins, G. M., Reinsel, G. C. & Ljung, G. M.*Time series analysis: forecasting and control* (John Wiley & Sons, 2015).

[24] Ester, M. *et al*. A density-based algorithm for discovering clusters in large spatial databases with noise. In *kdd*, vol. 96, 226–231 (1996).

[25] Page MJ (2021). The PRISMA 2020 statement: an updated guideline for reporting systematic reviews. Systematic reviews.

